# Creating Engineered NAC Flaps Using in Vivo Revascularization: A Proof-Of-Concept Study

**DOI:** 10.1007/s12015-026-11084-x

**Published:** 2026-02-19

**Authors:** Yanis Berkane, Alperen Abaci, Bradley W. Ellis, Loïc van Dieren, Edvin Hendi, Haizam Oubari, Nicolas Bertheuil, Ruben Oganesyan, Curtis L. Cetrulo, Michelle E. McCarthy, Mark A. Randolph, Alexandre G. Lellouch, Basak E. Uygun

**Affiliations:** 1 Center for Engineering in Medicine and Surgery, Massachusetts General Hospital, Harvard Medical School, Boston, MA 02114 USA; 2 Shriners Children’s Boston, Boston, MA 02114 USA; 3 Department of Plastic, Reconstructive and Aesthetic Surgery, CHU de Rennes, University of Rennes, Rennes, 35000 France; 4 SITI Laboratory, UMR1236, INSERM, Etablissement Francais du Sang, University of Rennes, Rennes, 35000 France; 5 Department of Pathology, Beth Israel Deaconess Medical Center, Harvard Medical School, Boston, MA 02215 USA; 6 Vascularized Composite Allotransplantation Laboratory, Center for Transplantation Sciences, Massachusetts General Hospital, Harvard Medical School, Boston, MA 02115 USA; 7 Department of Plastic and Reconstructive Surgery, Massachusetts General Hospital, Boston, MA 02114 USA; 8 Division of Plastic and Reconstructive Surgery, Cedars-Sinai Medical Center, Los Angeles, CA 90048 USA; 9 Department of General Surgery, Lahey Hospital and Medical Center, Burlington, MA 01805 USA; 10 Innovative Therapies in Hemostasis, INSERM UMR-S 1140, University of Paris, Paris, 75006 France

## Abstract

Acellular scaffold revascularization is the most significant challenge in tissue and organ engineering. To achieve successful and durable cell growth within complex three-dimensional scaffolds, a functional vascular network must ensure the supply of oxygen and nutrients within the matrix structure. Several teams have studied in vitro and ex vivo re-endothelialization using dynamic cell seeding and bioreactors, among other techniques. Still, to date, no approach has demonstrated long-term successful and functional vascular tree growth within 3D scaffolds. Herein, we propose a promising alternative using the in vivo implantation of decellularized nipple-areolar complex (NAC) scaffolds on a prepared vascular pedicle, thereby achieving engineered flap creation. We compared two implanted groups, one with and one without vascular micropunctures. After 3 weeks, explanted scaffolds demonstrated successful in-depth capillary growth, supported by contrast angiography, histology (including CD31 staining), and macroscopic bleeding. Flow cytometry assays identified different cell contingents, including CD45 and CD31-positive. No significant difference was found between the two implanted groups. This proof-of-concept study offers valuable insights into the successful engineering of composite tissues and organs.

**Graphical Abstract:**

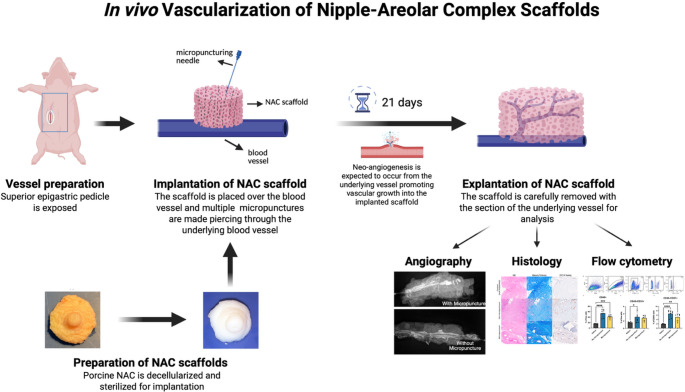

## Introduction

Breast cancer remains one of the leading cancer types worldwide, with approximately 4 million survivors in the United States alone and hundreds of thousands of new cases diagnosed annually [1,2,3]. With improving survival rates, the number of women undergoing mastectomy, and subsequent breast reconstruction, continues to increase. A crucial final step in the reconstructive journey is nipple–areolar complex (NAC) reconstruction, which has been shown to significantly enhance psychological well-being and provide patients with a sense of closure and completeness after cancer treatment [4, 5, 6, 7]. Commonly used reconstructive surgical techniques include local skin flaps, skin grafts from secondary donor sites, and three-dimensional tattooing [8, 9, 10, 11, 12, 13,14, 15, 16]. While these methods can recreate the visual appearance of the native NAC, their long-term outcomes are inconsistent and often fail to maintain nipple projection over time, frequently necessitating additional corrective procedures [17, 18, 19]. Various materials, such as cartilage, silicone gel, hyaluronic acid, and calcium hydroxyapatite, have been used in attempts to improve projection; however, issues such as depigmentation, skin flap necrosis, material degradation or modification, and loss of nipple projection have been widely reported, contributing to variable patient satisfaction [20, 21, 22].

To address the limitations of conventional NAC reconstruction methods, decellularized nipple scaffolds have emerged as a promising and innovative solution [23, 24, 25, 26]. Unlike synthetic scaffolds, these constructs are derived from native tissue and provide a non-immunogenic matrix with structural, mechanical, and biochemical complexity that is difficult to replicate using synthetic methods, and they have features essential for supporting functional tissue regeneration in the recipient [27, 28, 29]. By preserving the intricate extracellular matrix (ECM) architecture, decellularized scaffolds provide an optimal environment for reseeding with autologous cells and hold great potential for restoring both form and function in NAC reconstruction. To explore the potential of this approach, Pashos et al. utilized decellularized NACs derived from *rhesus macaques* [25]. In their in vivo model, the apical portion of decellularized nipples was removed and onlay grafted onto dermal beds, then explanted at 1-, 3-, and 6-weeks post-implantation. The results revealed evidence of neovascularization and endothelialization within the implanted tissues, indicating early stages of integration and regenerative potential. In another study, Oganesyan et al. employed decellularized swine nipples, which retained key ECM components and microarchitecture following decellularization [23]. The scaffolds demonstrated excellent biocompatibility both in vitro, when seeded with normal human dermal fibroblasts on the epithelial or dermal side, and in vivo, with no signs of inflammatory response observed 21 days after subcutaneous implantation.

Although decellularized nipple scaffolds hold great potential for creating anatomically and functionally realistic NAC models, achieving adequate vascularization at the implantation site remains a critical barrier to clinical success [30, 31, 32]. To address this, various pro-angiogenic strategies, such as growth factor delivery and cell incorporation, have been explored across different tissue types, although they often require several weeks to achieve effective vascular integration [33, 34, 35, 36, 37]. A recent and promising approach to promote angiogenesis is micropuncture-induced vascularization [38, 39]. This approach involves creating small, localized perforations in blood vessels to stimulate a pro-angiogenic environment, promoting rapid endothelial cell and macrophage infiltration into the scaffold. When applied to the rat femoral artery and vein, micropuncture significantly enhanced early vascular and immune cell infiltration compared to non-micropunctured controls [38]. However, this promising strategy has yet to be evaluated in the context of decellularized nipple scaffolds.

In this study, we present a novel strategy for NAC reconstruction by implanting decellularized NAC scaffolds onto a surgically prepared vascular pedicle to generate a vascularized NAC construct. To evaluate the potential of enhancing vascular integration, we compared two groups, with and without vascular micropuncture pretreatment. After three weeks, explanted scaffolds exhibited successful capillary ingrowth, as confirmed by contrast angiography and histological analysis. Flow cytometry further identified key infiltrating cell populations, including CD45 + and CD31 + cells. This proof-of-concept study offers important insights into engineering vascularized composite tissues for future applications in NAC reconstruction.

## Materials and Methods

### Animals and Nipple Procurement

NACs were procured from deceased 30-kg female Yorkshire pigs following terminal procedure within Knight Surgery Research Laboratories (Massachusetts General Hospital, Harvard Medical School, Boston, MA). All postmortem procurements were approved by the local IACUC. Circular incisions were performed to harvest 30–40 mm diameter NACs in full thickness using a 15-scalpel blade. The NACs were then thinned using curved Mayo scissors, to remove all subcutaneous tissue and excess gland, achieving a flattened deep surface. The NACs were then placed in gauze and saline solution and transported to the bench for scaffold preparation.

### Scaffold Preparation

Porcine nipples measuring 30–40 mm were decellularized using our previously established protocol [23]. Initially, the tissues were rinsed in sterile phosphate-buffered saline (PBS) for 30 min, followed by a 12-hour wash in deionized water. They were then incubated in 0.2% (w/v) sodium dodecyl sulfate (SDS) for 48 h. Next, the nipples were treated with a deepithelialization solution containing 0.5 M sodium chloride (NaCl), 0.5% SDS, 0.25% ethylenediaminetetraacetic acid (EDTA), and 50 mM Tris buffer. This step was followed by immersion in 2 M NaCl for 24 h at room temperature, after which the tissue was manually deepithelialized. Subsequently, the samples underwent a series of detergent treatments: 0.2% SDS for 72 h, 0.5% SDS for 24 h, and 1% Triton X-100 for 48 h. They were then rinsed with PBS for 6 h, followed by 1-hour enzymatic treatment in a solution containing 1 mg/mL DNase I and 5 mM Mg²⁺ to promote enzymatic activity. Finally, the decellularized nipple scaffolds were thoroughly washed in PBS overnight and stored at 4 °C in PBS supplemented with 2% penicillin-streptomycin until further use. Unless otherwise specified, all steps were carried out at room temperature with continuous agitation at 240 rpm.

### DNA, Collagen, Glycosaminoglycans, and Growth Factor Analysis

DNA was extracted from both native and decellularized tissues using the DNeasy Blood & Tissue Kit (Qiagen, Germantown, MD, USA), following the manufacturer’s instructions on approximately 25 mg of tissue per sample. Briefly, tissue samples were incubated overnight at 56 °C in a proteinase K solution to ensure complete digestion. After the addition of buffer and ethanol, the mixture was transferred to a spin column, and repeated elutions were performed. Purified DNA from each sample was quantified using a NanoDrop spectrophotometer (260 nm; Thermo Fisher Scientific, Waltham, MA, USA) following the manufacturer’s protocol.

Similarly, total collagen content was measured using the Total Collagen Kit (QuickZyme Biosciences, Leiden, The Netherlands). Quantification of glycosaminoglycans (GAGs) was performed using a Total Glycosaminoglycans Assay Kit (Colorimetric, ab289842, Abcam Limited, Cambridge, UK).

Growth factor analysis was performed using the RayBio^®^ Human Growth Factor Antibody Array G-Series 1 kit (RayBiotech Life, Peachtree Corners, GA, USA) following the manufacturer’s recommendations.

### In Vivo Implantation and Experimental Groups

The objective was to isolate an arteriovenous pedicle to put in contact with the implanted scaffolds. A 30-kg Yorkshire female pig was used as the recipient. All survival and terminal procedures were approved by the local IACUC under protocol 2019N000179. The procedure was performed under general anesthesia and orotracheal intubation. Following skin preparation, two 10-cm paramedian incisions were made using a 15-scalpel blade, and electric cautery was used for subcutaneous and initial muscle dissection. Once entering the rectus abdominis muscles, intramuscular dissection was pursued using Metzenbaum scissors until exposing the superior epigastric pedicle (one artery and two veins on each side). The dissection pocket was completed, and collateral branches were ligated to obtain a 12-cm clean dissection of the vascular pedicle. On the right side, the vessels underwent 25 micropunctures using an 8 − 0 suture needle (**Micropuncture group**). On the left side, no punctures were performed (**No micropuncture group**). Four NAC scaffolds were placed in each pocket, immediately above the underlying superior epigastric vessels, with the flat/deep part of the NACs in contact with the vessels. The scaffolds were then secured to the underlying fascia using interrupted 4 − 0 Vicryl sutures. Following careful cauterization of potential bleeding, the dissection pockets were rinsed using 50 mL of sterile saline and a three-layer closure was performed using 3 − 0 absorbable poliglecaprone (interrupted inverted stitches and intradermal running suture). Sterile gauze and transparent adhesive dressings were used to protect the surgical approach. One intraoperative IV flash and daily postoperative IM injections of cotrimoxazole were administered to prevent postoperative infection. Pain management was carefully addressed in accordance with the IACUC-approved protocol. The animal was monitored daily for 3 weeks before the second procedure, with free access to food and water.

Control groups consisted of non-implanted decellularized NACs (**Decellularized group**) and fresh untreated NACs (**Native group**).

### Macroscopic examination, Angiography and Sample Preparation

Twenty-one days after implantation, the animal underwent a second general anesthesia similar to the implantation procedure. The previous scars were reused to reopen the implantation pockets, revealing the four NACs on each side. An *en bloc* dissection was performed to isolate the four NACs and the underlying pedicle as a single block. The pedicle was then ligated distally. Following macroscopic examination, the proximal side of the vessels was ligated and divided, freeing the engineered vascularized flap. An 18-G catheter was inserted into the arteries and secured using a 3 − 0 silk tie. Backtable angiography was performed by injecting 10 cc of 300 mg/ml Iohexol contrast agent and X-Ray. The recipient was then euthanized using a single IV injection of 100 UI pentobarbital. Other teams were able to harvest post-mortem organs and tissue as per protocol.

Following angiography, the 8 NACs were separated from the vessels and isolated. The infected NAC was discarded. Each implant was divided into three equal parts from the tip of the nipple. One third was immediately fixed in 10% buffered formaldehyde, one third was sent to the bench for cell isolation, and one third was snap-frozen in liquid nitrogen.

### Cell Isolation and Flow Cytometry Analysis

#### Native and Revascularized NAC Cell Isolation

NACs (either native or revascularized) were procured in a sterile fashion and submerged in ice-cold Hank’s Balanced Salt Solution (HBSS, Thermo Fisher, Waltham, MA, USA) and transferred to a sterile biosafety hood. The NACs were then minced and digested in a solution of collagenase type IV (1 mg/mL, MilliporeSigma, Burlington, MA, USA) and dispase (1.2 U/mL, Stemcell Technologies, Vancouver, BC, Canada) in HBSS at 37 °C for 1.5 h with gentle agitation. After digestion, two volumes of Dulbecco’s Modified Eagle Medium (DMEM, Thermo Fisher, Waltham, MA, USA) supplemented with 10% fetal bovine serum (FBS, Peak Serum, Inc., Erie, CO, USA), 1% glutamine (Gibco, Thermo Fisher Scientific, Waltham, MA, USA), and 1% P/S (Invitrogen, Thermo Fisher Scientific, Waltham, MA, USA) was added to the solution and the cells were filtered through a 100 μm cell strainer. After filtration, the cells were spun down at 1,100 x g for 10 min with no break and then resuspended in a solution of supplemented DMEM and Red Blood Cell Lysis (Invitrogen, Thermo Fisher Scientific, Waltham, MA, USA) at a 1:7 ratio. The cells were then filtered through a 40 μm cell strainer and spun down at 1,100 x g for 10 min with no break. The cells were then resuspended in a freezing medium consisting of 90% supplemented DMEM and 10% dimethyl sulfoxide (DMSO, MilliporeSigma, Burlington, MA, USA) and cryopreserved in liquid nitrogen until further analysis.

#### Pig Aortic Endothelial Cell Isolation

A small section of the descending aorta was excised in a sterile fashion and submerged in ice-cold HBSS and transferred to a sterile biosafety hood. The vessel was then cut into 1 cm x 1 cm sections and placed in a petri dish with the endothelial cell layer facing down and cultured in endothelial growth medium 2 (EGM, Lonza, Walkersville, MD, USA) with media changes every other day until endothelial sprouting was observed. Once sprouting was observed, the tissues were removed, and the endothelial cells were detached using trypsin (Thermo Fisher, Waltham, MA, USA), spun down at 1,100 x g for 5 min with low break and seeded into a T-25 flask (Celltreat, Pepperell, MA, USA) and cultured to 70% confluency. After reaching 70% confluency, the cells were then passaged into a T-75 flask and cultured to 70% confluency and then passaged into 3 T-75 flasks. Once the flasks reached 70% confluency, the cells were detached, spun down, and resuspended in freezing media consisting of 90% EGM and 10% DMSO and cryopreserved in liquid nitrogen until further analysis. These cells were used to validate the CD31 antibodies used for flow cytometry analysis.

Flow cytometry was performed using a Cytek Northern Lights spectral cytometer. Cells were first incubated with eBioscience™ Fixable Viability Dye eFluor™ 506 (1:1000; Thermo Fisher, Waltham, MA, USA) in PBS without calcium or magnesium for 30 min at 4 °C in the dark to assess viability. Cells were then stained with surface antibodies, including anti-CD45 (clone K252.1E4, 1:10; Bio-Rad, Hercules, CA) and anti-CD31 (clone LCI-4, 1:10; Bio-Rad, Hercules, CA), for 30 min in FACS medium under light-protected conditions at 4 °C. Samples were immediately acquired on the cytometer, and data was analyzed using OMIQ software (Dotmatics, Woburn, MA, USA).

### Histology and Immunohistochemistry

The tissue samples were fixed with 10% neutral buffered formalin for 24 h, dehydrated, embedded in paraffin, microsectioned at 5 μm, and then stained with hematoxylin and eosin (H&E), Masson trichrome, Alcian blue, and CD31 (ab28364, Abcam, Cambridge, MA, USA). The slides were then scanned using a NanoZoomer S360MD (Hamamatsu Photonics, Hamamatsu, Japan). Conventionally stained (H&E, Masson trichrome, Alcian blue) slides were reviewed independently by two histopathology trained researchers, whereas the immunohistochemical stain (CD31) was assessed quantitatively by a pathologist.

All images were of intact cross-sections and were subjected to manual quality check before being exported in NDPI format for image analysis applications. Quantitative image analysis was performed using QuPath software (version 5.1.0 [40]). To eliminate inter-operator subjectivity, a single operator (RVO) performed all the software analyses. First, the whole section area was manually annotated and measured (µm^2^). Next, the total number of cell nuclei was measured for each image using the “Cell detection” command. Then the CD31 positive cells were selected and classified as such using the “Single measurement classifier” command, DAB channel filter, and “Nucleus: DAB OD Mean” measurement. After that, the total number of CD31-positive cell nuclei was normalized to the whole analyzed area, resulting in positive nuclei/µm^2^ values. Supervised digital segmentation was employed to add missing objects or eliminate incorrectly identified ones, with supervised corrections accounting for less than 10% of objects per image.

### Statistical Analysis

Experiments were conducted with a minimum of three replicates. Data are reported as mean ± standard error of the mean (SEM). Statistical significance was assessed using a two-tailed student’s t-test for DNA, collagen, and GAG content; two-way ANOVA for growth factor analysis; and one-way ANOVA for CD31 comparison, with a p-value of ≤ 0.05 considered statistically significant. Analyses were performed using Prism (v. 10.3.0, GraphPad, La Jolla, CA, USA).

## Results

### Efficiency of Decellularization Protocol

**Fig. 1:**
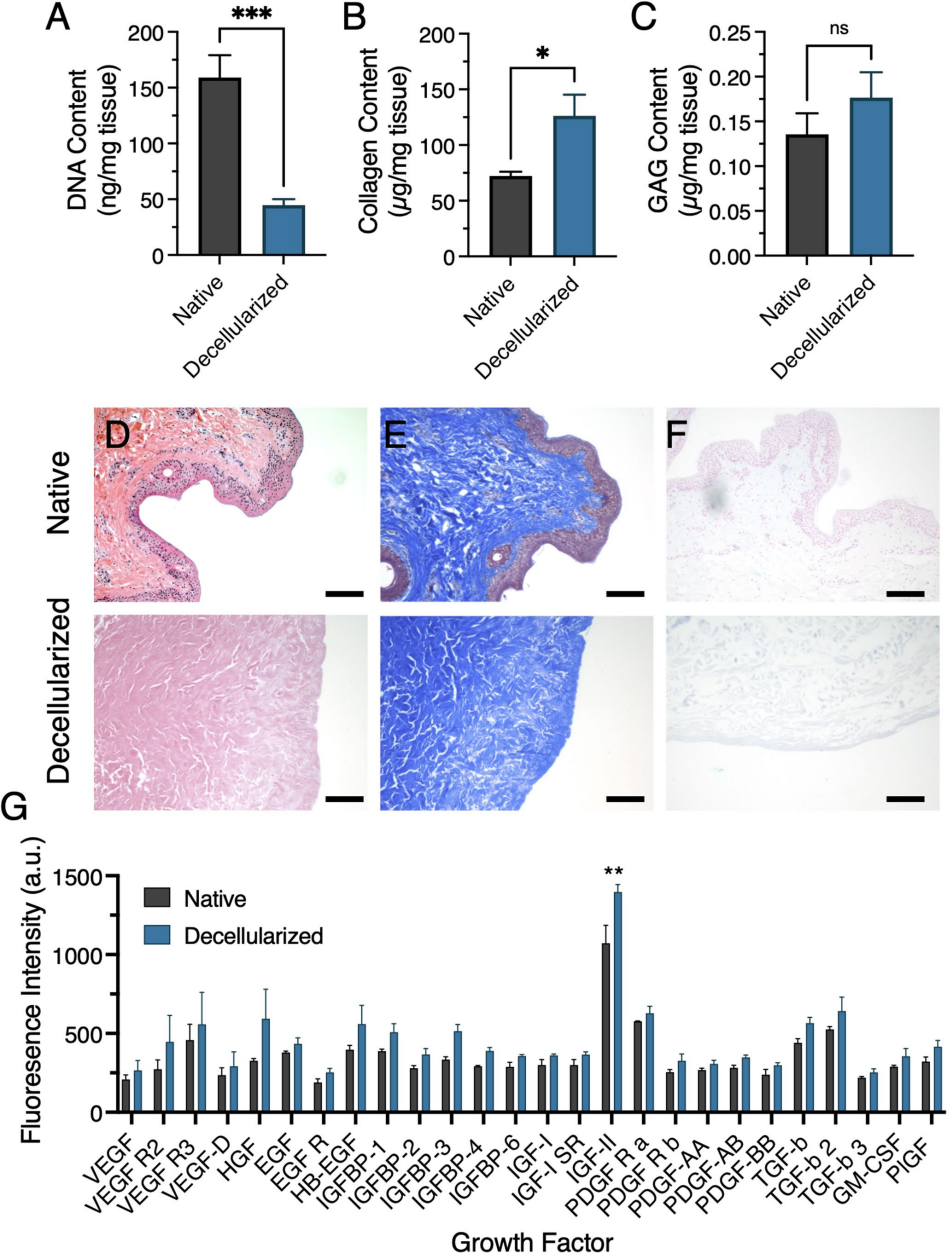
Assessment of decellularization of porcine NAC scaffolds. Quantification of (**A**) DNA (*n* = 5), (**B**) collagen (*n* = 3), and (**C**) GAG (*n* ≥ 4) content in native and decellularized NACs. Representative histological sections of native and decellularized NACs stained with (**D**) H&E, (**E**) Masson’s Trichrome, and (**F**) Alcian Blue. Scale bars: 250 μm. (**G**) Retention of growth factor content in native and decellularized NACs (*n* = 3). Data are presented as mean ± standard error of the mean. ***p < 0.001, **p < 0.01, ns: *p* > 0.05

To evaluate the efficiency of DNA removal, tissue sections from five different native and decellularized nipples were analyzed for DNA content. Following a series of chemical and enzymatic treatments, the DNA content was significantly reduced, from 159.0 ± 20.1 ng DNA/mg wet tissue in native samples to 44.8 ± 5.3 ng DNA/mg wet tissue in decellularized tissues, indicating substantial removal of nuclear material with statistical significance **(**Fig. 1A**)**. Additionally, a marked visual loss of tissue pigmentation served as an immediate, qualitative indication of successful decellularization. In addition to confirming the removal of cellular material from the tissues, quantitative analysis of key ECM components showed that both collagen and GAG content were well preserved following decellularization. Specifically, collagen levels were 72.2 ± 3.9 µg/mg wet tissue before and 126.3 ± 19.0 µg/mg after decellularization **(**Fig. 1B**)**, while GAG levels were 0.14 ± 0.02 µg/mg wet tissue before and 0.18 ± 0.03 µg/mg tissue after **(**Fig. 1C**)**, with no loss observed in the key ECM components.

Following quantitative confirmation of decellularization efficacy, histological assessment of the decellularized nipples using H&E **(**Fig. 1D**)** staining confirmed effective removal of cellular components while retaining a substantial amount of ECM. Beyond confirming cell removal, staining with Masson’s Trichrome **(**Fig. 1E**)** and Alcian Blue **(**Fig. 1F**)** demonstrated that key ECM components, collagen and GAGs, were successfully preserved.

In addition to histological and biochemical assessment of ECM components, the retention of key growth factors is a critical consideration when evaluating decellularization protocols. To assess this, we analyzed native and decellularized NACs using a growth factor array and found no significant change in angiogenesis-related factors (Vascular Endothelial Growth Factor (VEGF), Hepatocyte Growth Factor (HGF), Epidermal Growth Factor (EGF), Insulin-like Growth Factor (IGF), and Platelet-Derived Growth Factor (PDGF)) besides IGF-II, or factors involved in cell recruitment and matrix integration during wound repair (Transforming Growth Factor Beta (TGF-β), Granulocyte-Macrophage Colony-Stimulating Factor (GM-CSF), and Placental Growth Factor (PlGF)) **(**Fig. 1G**)**. These findings suggest that the decellularization process effectively preserves essential bioactive molecules that support revascularization and tissue regeneration.

### Macroscopic and Angiography Analysis

**Fig. 2:**
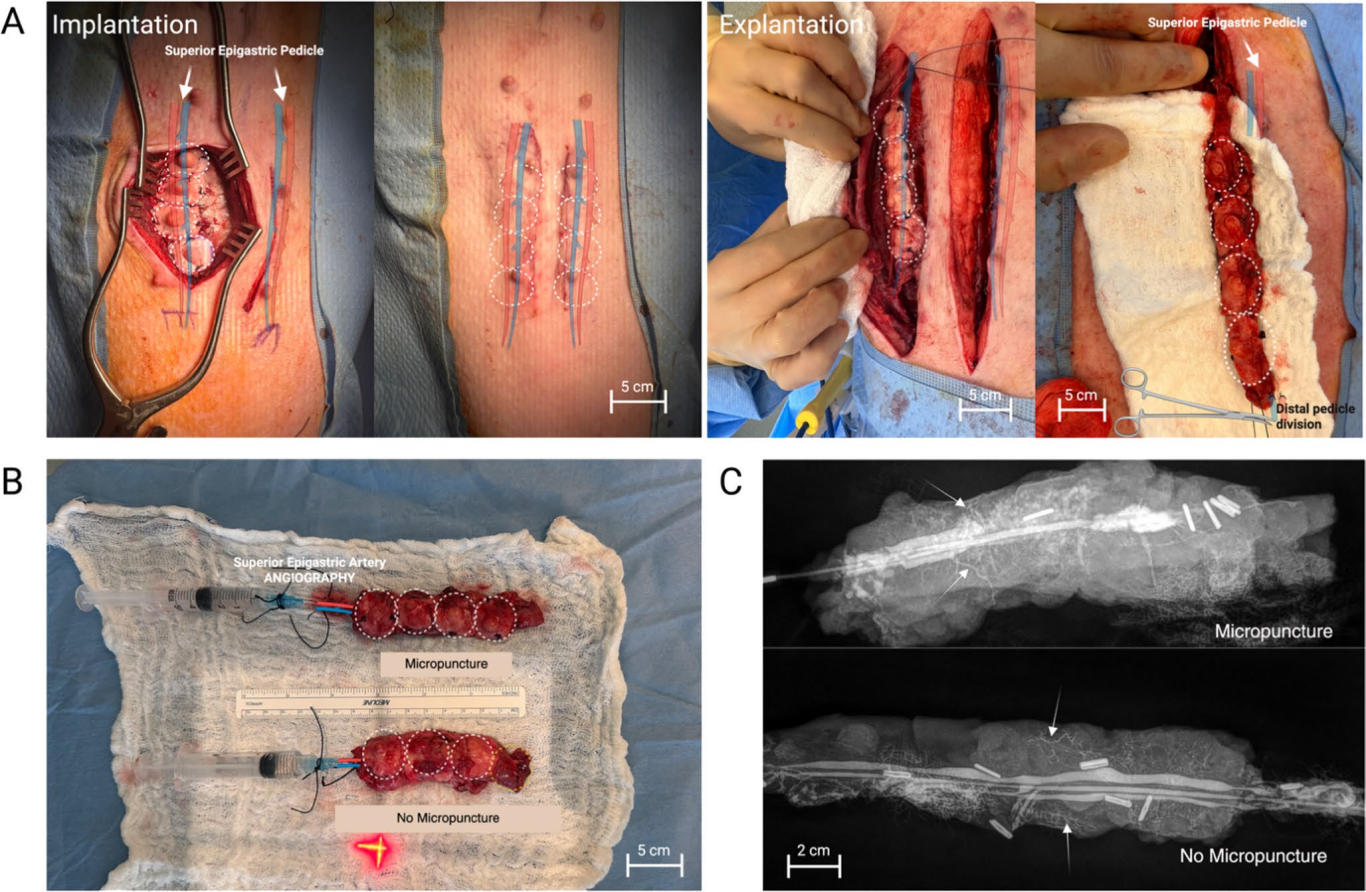
In vivo assessment of porcine NAC scaffold for vascularization. (**A**) Implantation and explantation procedures. (**B**) Macroscopic and (**C**) X-ray imaging of the explanted NACs from micropuncture and no micropuncture groups, with the contrast agent injected into the vasculature Dashed white circles define the NAC boundaries for easy identification

The decellularized NACs were implanted over micropuncture or non-micropuncture blood vessels and kept for 21 days. Following *en bloc* isolation and distal pedicle division at the end of 3 weeks, bleeding on the surface of the NACs was observed in both groups, and pulsatile flow was preserved within the superior epigastric vessels, indicating successful integration of the implants and vascular patency **(**Fig. 2A**)**. No qualitative macroscopic difference was found between the two implanted groups **(**Fig. 2B**)**. No hematoma was found in either group, which was consistent with the absence of intraoperative bleeding. Contrast angiography revealed successful complete opacification of the epigastric vessels (artery and vein), demonstrating a closed loop. Intra-scaffold anastomotic bridges were confirmed through capillary-like opacification, demonstrating successful inosculation/neovascularization **(**Fig. 2C**)**. No difference was noted between groups in contrast intake, and no quantitative analysis was performed. One NAC complex in the micropuncture group showed signs of infection and was discarded.

### Histological Analysis

**Fig. 3:**
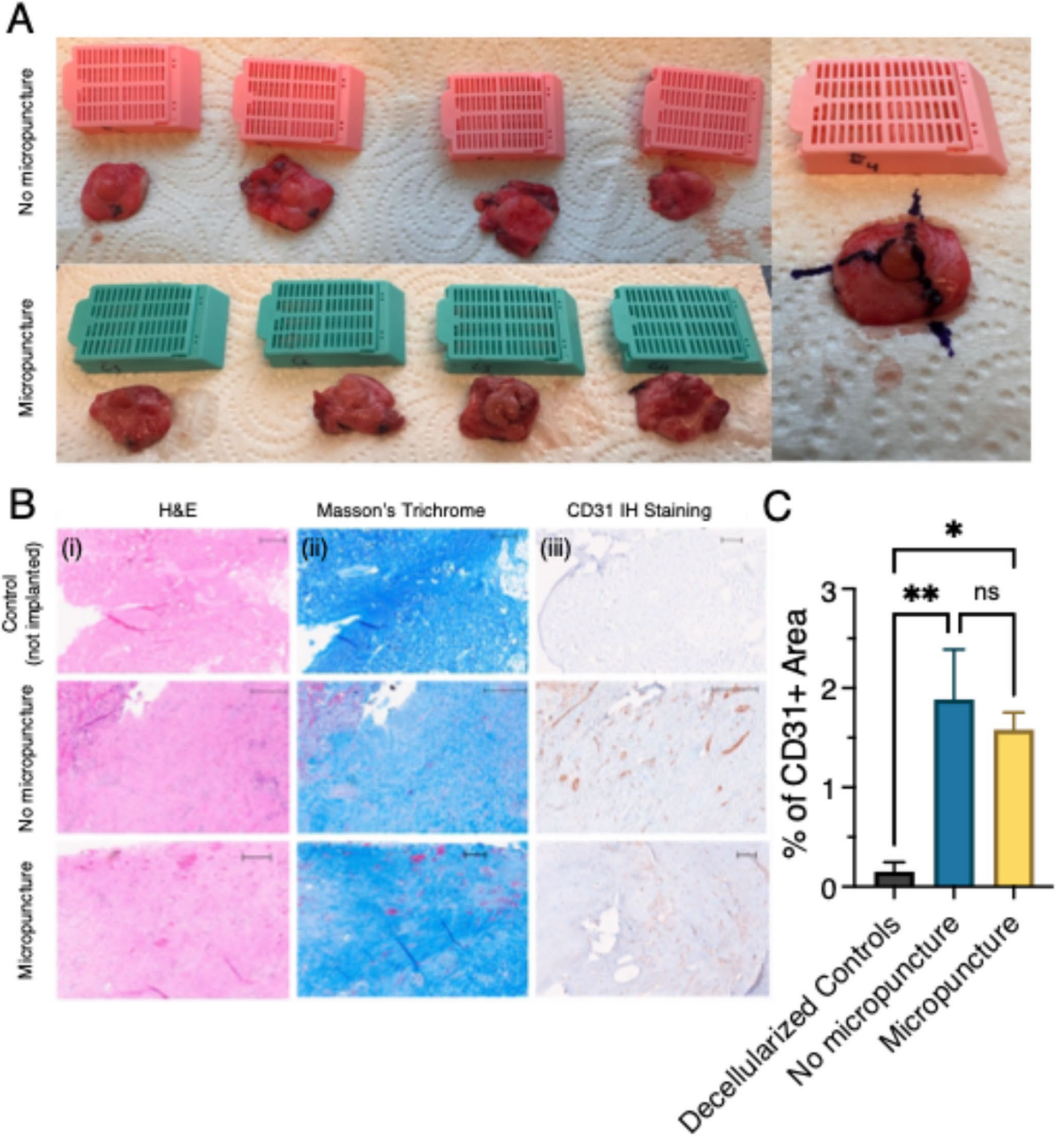
Analysis of implanted NAC scaffolds. (**A**) Tissue preparation for histology. (**B**) Histological assessment of explanted NACs, as well as non-implanted decellularized controls by H&E, Masson’s Trichrome and CD31 IH staining. Scale bars: 300 μm. (**C**) Percentage of CD31 positive areas in the explanted NACs and decellularized controls. **p < 0.01, *p < 0.05, ns: *p* > 0.05

After explantation, the NACs were processed for histological analysis **(**Fig. 3A**)**. Both implanted samples exhibited increased cellularity and tissue infiltration **(Fig. 3Bi)** when compared to H&E-stained sections of the decellularized control group. Masson’s Trichrome staining further demonstrated connective tissue formation in implanted NACs, which was notably lower in non-implanted controls **(Fig. 3Bii)**. CD31 immunostaining revealed significant endothelialization in both implanted groups **(Fig. 3Biii)**. Quantitative analysis revealed higher CD31 expression in the No Micropuncture (1.86 ± 0.50%) and Micropuncture (1.58 ± 0.17%) groups compared to non-implanted controls (0.15 ± 0.10%), with no significant difference between the two implanted groups (*p* = 0.7446) **(**Fig. 3C**)**. These findings suggest that decellularized nipple scaffolds support vascularization upon implantation, but micropuncture does not significantly enhance this angiogenic response.

### Flow Cytometry Analysis

**Fig. 4:**
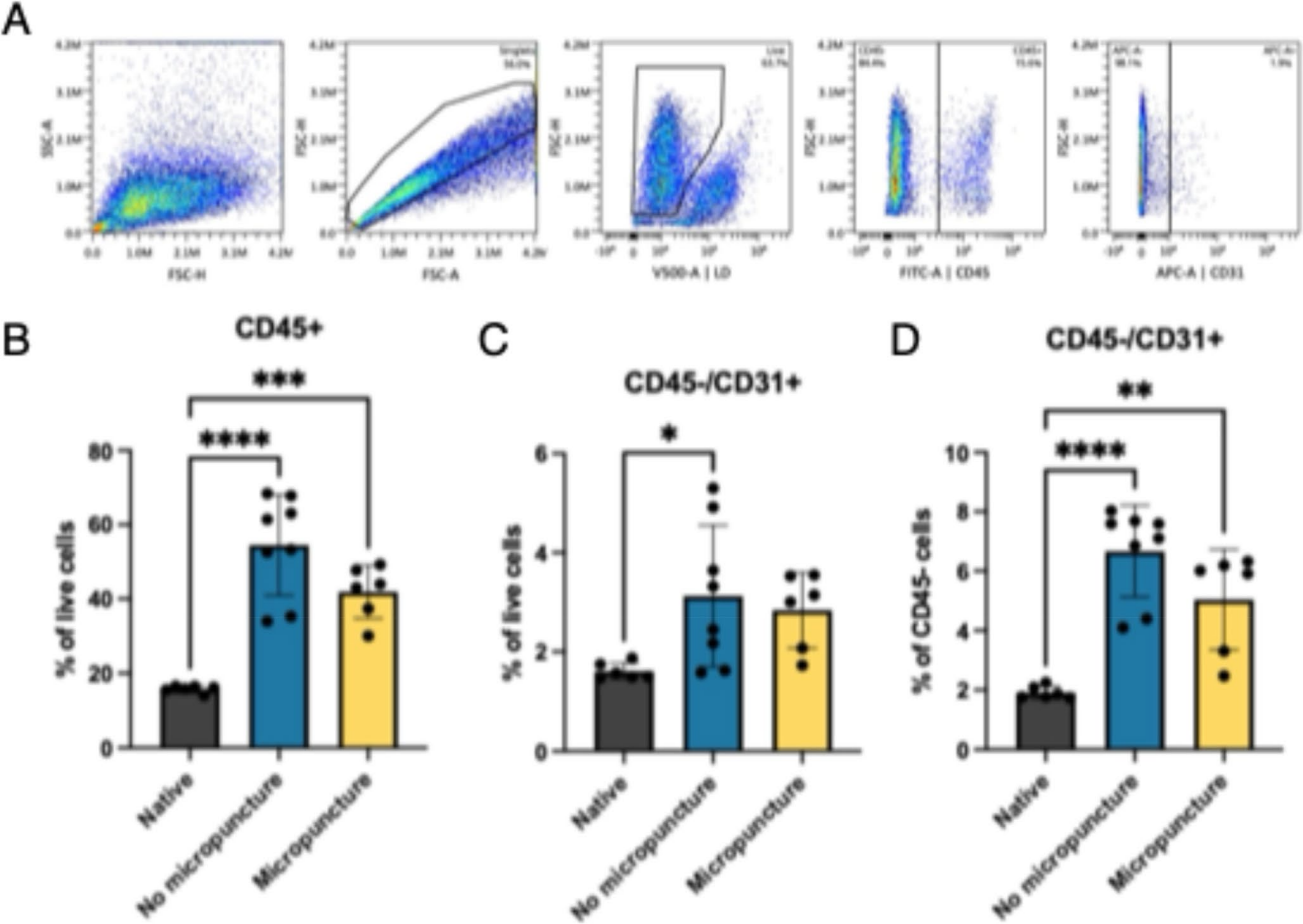
Flow cytometry analysis of native and decellularized NACs. (**A**) Gating strategy. (**B**) CD45 + cells as a percentage of live cells. C&D) CD45-/CD31 + cells as a percentage of live cells (**C**) or CD45- cells **D**). ****p < 0.0001, ***p < 0.001, **p < 0.01, *p < 0.05

Flow cytometry analysis (Fig. 4) showed that CD45⁺ cells constituted 15.8% ± 0.9 of live cells in native nipples, 54.5% ± 13.6 in the “no micropuncture” group, and 41.9% ± 7.1 in the micropuncture group **(**Fig. 4B**)**. CD45^−^/CD31⁺ cells accounted for 1.6% ± 0.2 of live cells in native nipples, 3.1% ± 1.4 in the “no micropuncture” group, and 2.8% ± 0.8 in the micropuncture group **(**Fig. 4C**)**. Within the CD45⁻ compartment, CD31⁺ cells represented 1.9% ± 0.2 in native nipples, 6.7% ± 1.5 in the “no micropuncture” group, and 5.0% ± 1.7 in the micropuncture group **(**Fig. 4D**)**.

## Discussion

In this study, we evaluated an in vivo scaffold revascularization by promoting angiogenesis from isolated vessels during implantation of NAC scaffolds. The impact of preliminary vascular micropunctures was also studied. Our findings demonstrated that both surgical approaches enhanced the migration of endothelial cells into the decellularized grafts and formation of capillary-like structures consistent with neovascularization, as supported by in situ bleeding, positive scaffold angiography, and CD31 immunohistochemistry. We did not detect a statistically significant difference attributable to micropuncture at the 3-week endpoint.

We previously validated a decellularization protocol that ensures efficient removal of cells while preserving the ECM components, with only minor alterations in composition, both quantitatively and qualitatively [23]. Our results presented here confirmed the efficacy of our approach further by quantitative and qualitative analysis of ECM components. Tissue harvesting and sampling procedures may have slightly differed between studies, explaining minor changes in ECM and total tissue DNA content, but no changes were made to the decellularization protocol, ensuring adequate reduction in DNA and optimal preservation of the ECM. However, beyond maintaining the structural and biochemical integrity of the ECM, the retention of growth factors is also critical for successful tissue revascularization and regeneration. Growth factors such as VEGF, HGF, EGF, IGF, PDGF, TGF-β, GM-CSF, and PlGF play essential roles in promoting angiogenesis by regulating cell proliferation, differentiation, survival, and migration, ultimately facilitating the formation of new blood vessels at the implantation site [41, 42, 43, 44]. Importantly, our data indicated that these key growth factors were well preserved and not significantly reduced following decellularization. This finding aligns with the study by Pashos et al., who reported that their decellularized nipples derived from non-human primates retained proteins that support cell migration, adhesion, differentiation, and promote both re-epithelialization and revascularization [25]. In contrast, Maistriaux et al. observed a substantial loss of growth factors in human NACs decellularized using chemical and enzymatic treatments [45]. Although similar decellularization agents were used in their protocol compared to ours, differences in reagent concentrations and the source of donor tissue likely contributed to the observed discrepancies in growth factor retention. Besides decellularized NACs, comparable preservation of growth factors has also been reported in the decellularization of various vascularized composite allografts, including facial, penile, auricular, and cutaneous flaps [46, 47, 48, 49, 50]. These findings further support our results, suggesting that, although complete retention may not always be possible, most critical angiogenic factors can be maintained with optimized decellularization methods.

Recellularization of decellularized tissues is a crucial step in restoring function to biologically derived scaffolds. By repopulating these acellular matrices with appropriate cell types, the aim is to develop functional, biocompatible tissues capable of integrating with host systems for regenerative medicine applications. This strategy has also been applied to the NAC, where various cell types have been used to evaluate the in vitro recellularization capacity of decellularized NAC scaffolds. Pashos et al. demonstrated that bone marrow-derived stem cells seeded onto decellularized NACs exhibited high viability (> 90%) and actively migrated within the scaffold [51]. Similarly, Oganesyan et al. [23] and Maistriaux et al. [45] showed that human fibroblasts were shown to attach, grow on, and infiltrate the decellularized NACs. Supporting this finding, previous studies have highlighted the regenerative potential of decellularized NAC scaffolds. For instance, researchers demonstrated host-driven neovascularization and re-epithelialization following onlay transplantation of decellularized NAC grafts in both murine [52] and rhesus macaque non-human primate (NHP) models [53]. Moreover, the NHP models exhibited evidence of innervation alongside robust vascular and epithelial coverage. Complementary studies have also highlighted the biocompatibility and recellularization capacity of decellularized nipple scaffolds [23, 26, 45]. Together, these findings reinforce the rationale for using decellularized nipple tissue in regenerative applications, offering native ECM architecture and preserved growth factors at the implantation site. While NAC scaffolds can be successfully reseeded with cells, complete scaffold revascularization remains a critical prerequisite, as vascularization is essential for supporting long-term cell survival, tissue integration, and healing. To address this, many researchers have explored in vitro and ex vivo re-endothelialization approaches, which often require complex strategies involving surface modification, optimized culture conditions, and the use of multiple cell types [54, 55, 56, 57, 58, 59]. In this study, we developed an alternative in vivo revascularization strategy to support the survival of the implanted tissue in the long term. Decellularized NACs were implanted in direct contact with the arterial-venous pedicle to promote angiogenesis into the scaffolds. We did not observe macroscopic signs of inflammation, rejection, or graft failure; one scaffold was excluded for localized infection as stated in the Results. Consistent with these macroscopic findings, histology did not demonstrate features of acute or chronic inflammatory tissue damage, fibrosis or necrosis. Flow cytometry revealed a higher proportion of CD45⁺ leukocytes in implanted scaffolds compared with native tissue, whereas CD31⁺ endothelial cells remained a minority population. We interpret this leukocyte enrichment as a hallmark of constructive host remodeling rather than pathologic inflammation or rejection, in line with prior reports across decellularized scaffold models in large animals and primates, where early CD45⁺ infiltration marks matrix remodeling, angiogenic signaling, and scaffold integration (e.g., heart valve and lung scaffolds) [60, 61, 62]. Moreover, work from Badylak’s group has shown that decellularized matrices can polarize macrophages from an M1 to M2 phenotype, supporting a pro-remodeling milieu rather than persistent inflammation [63, 64]. We also note an important methodological consideration: enzymatic dissociation for flow cytometry can under-recover endothelial cells tightly integrated within vascular structures and ECM, biasing against CD31⁺ detection. This helps explain the apparent discrepancy between the modest CD31⁺ fraction by flow cytometry and the clear endothelialization observed by tissue-level assessments (CD31 immunohistochemistry and angiography). Future studies will further investigate the CD45 + population to establish the M1-to-M2 phenotype conversion and to provide more detailed subpopulations using advanced cytometry.

To explore the alternative technique of in vivo revascularization, we leveraged well-established plastic and reconstructive surgery principles. The first of these is autonomization, also known as inosculation, where the vascular network in the vascularized flap connects with the recipient site’s network by creating connections with the wound bed and dermal margins [65, 66, 67]. This principle has been used for centuries in temporarily pedicled flap techniques [68, 69, 70]. Another cornerstone of this proof-of-concept is prefabricated flaps. In some studies, authors have described the creation of newly vascularized anatomical units by placing vessels beneath targeted skin areas, enabling *en-bloc* dissection following a delay for neo-vascularization between the vessels and the neo-flap [71, 72]. Finally, a third innovative method is vascular micropuncture, which involves creating microscopic, controlled perforations in recipient blood vessels using ultrafine needles [38, 39]. This technique potentially accelerates vascularization by facilitating rapid cellular extravasation and capillary formation within the implanted scaffold. Hancock et al. indeed demonstrated that collagen scaffolds placed over micropunctured vessels in a rat model showed significantly greater cellular infiltration, including endothelial cells (CD31) and macrophages (F4/80), compared to non-micropunctured controls [38]. In our study, we combined all three principles and applied them to NAC scaffolds. By placing the decellularized NACs in contact with a prepared arteriovenous pedicle, we created an engineered flap construct through neovascularization from the vessels to the matrix. After 21 days of implantation, both groups, with and without micropuncture, exhibited early signs of revascularization, confirmed through angiography, histology, immunohistochemistry, and flow cytometry. Both conditions supported robust tissue formation and cellular repopulation, but micropuncture did not appear to further enhance vascularization. This may be due to the intrinsic regenerative properties of decellularized nipple scaffolds, suggesting that additional surgical intervention may not be necessary for effective graft integration. Another explanation could be the lack of statistical power due to the 3-week delay, which might be sufficient to enable neovascularization even with no micropuncture enhancement. Earlier timepoint assessments could help address this theory. Despite comparable profiles in endothelial cell content and immune cell profile between groups, no analysis of angiogenic factors was performed in the explanted scaffolds, which should be explored. Finally, despite promising results, this proof-of-concept study presents obvious power limitations, and further studies should confirm these preliminary outcomes and lead to further optimization of in vivo strategies for acellular scaffold revascularization.

## Conclusion

In summary, this study suggests a novel approach to NAC reconstruction by implanting decellularized NAC scaffolds onto a surgically prepared vascular pedicle, creating a vascularized tissue-engineered flap. Vascular micropuncture pretreatment was evaluated as a strategy to enhance vascular integration, with comparative analysis between treated and untreated groups. After three weeks, capillary ingrowth was observed in the explanted scaffolds, as confirmed by contrast angiography and histological evaluation. Flow cytometry also revealed key infiltrating cell populations, including CD45 and CD31-positive cells. These findings support the feasibility of engineering vascularized composite tissues and provide a foundation for future advancements in NAC reconstruction.

## Data Availability

All raw data is available upon reasonable request to the corresponding authors.
